# Osseous Union after Mandible Reconstruction with Fibula Free Flap Using Manually Bent Plates vs. Patient-Specific Implants: A Retrospective Analysis of 89 Patients

**DOI:** 10.3390/curroncol29050274

**Published:** 2022-05-06

**Authors:** Michael Knitschke, Sophia Sonnabend, Fritz Christian Roller, Jörn Pons-Kühnemann, Daniel Schmermund, Sameh Attia, Philipp Streckbein, Hans-Peter Howaldt, Sebastian Böttger

**Affiliations:** 1 Department of Oral and Maxillofacial Surgery, Justus-Liebig-University, Klinikstrasse 33, 35392 Giessen, Germany; sophia.m.sonnabend@dentist.med.uni-giessen.de (S.S.); daniel.schmermund@uniklinikum-giessen.de (D.S.); sameh.attia@dentist.med.uni-giessen.de (S.A.); philipp.streckbein@uniklinikum-giessen.de (P.S.); hp.howaldt@uniklinikum-giessen.de (H.-P.H.); sebastian.boettger@uniklinikum-giessen.de (S.B.); 2 Department of Diagnostic and Interventional Radiology and Pediatric Radiology, Justus-Liebig-University, Klinikstrasse 33, 35392 Giessen, Germany; fritz.c.roller@radiol.med.uni-giessen.de; 3 Institute of Medical Informatics, Justus-Liebig-University Giessen, 35392 Giessen, Germany; joern.pons@informatik.med.uni-giessen.de

**Keywords:** osseous free flaps, patient-specific implants, plate-related complications, free fibula flap, bone healing, osseous union, ossification

## Abstract

The aim of this monocentric, retrospective clinical study was to evaluate the status of osseous union in uni- and poly-segmental mandible reconstructions regarding conventional angle-stable manually bent osteosynthesis plates (Unilock 2.0 mm) versus titan laser-melted PSI patient-specific implant’s (PSI). The clinical impact of PSI’s high stiffness fixation methods on bone healing and regeneration is still not well addressed. The special interest was in evaluating the ossification of junctions between mandible and fibula and between osteotomized fibula free flap (FFF) segments. Panoramic radiograph (OPT), computed tomography (CT) scans, or cone-beam CTs (CBCT) of patients who underwent successful FFF for mandible reconstruction from January 2005 to December 2020 were analyzed. A total number of 89 cases (28 females (31.5%), 61 males (68.5%), mean age 58.2 ± 11.3 years, range: 22.8–82.7 years) fulfilled the chosen inclusion criteria for analysis (conventional: *n* = 44 vs. PSI: *n* = 45). The present study found an overall incomplete ossification (IOU) rate of 24.7% (conventional: 13.6% vs. PSI: 35.6%; *p* = 0.017) for mandible to fibula and intersegmental junctions. Between osteotomized FFF segments, an IOU rate of 16% was found in the PSI-group, while no IOU was recorded in the conventional group (*p* = 0.015). Significant differences were registered for IOU rates in poly-segmental (*p* = 0.041), and lateral (*p* = 0.016) mandibular reconstructions when PSI was used. Multivariate logistic regression analysis identified plate exposure and type of plate used as independent risk factors for IOU. Previous or adjuvant radiotherapy did not impact incomplete osseous union in the evaluated study sample. PSI is more rigid than bent mini-plates and shields functional mechanical stimuli, and is the main reason for increasing the rate of incomplete ossification. To enhance the functional stimulus for ossification it has to be discussed if patient-specific implants can be designed to be thinner, and should be divided into segmental plates. This directs chewing forces through the bone and improves physiological bone remodeling.

## 1. Introduction

Jaw reconstruction with a free fibula flap (FFF) has been raised to today’s gold standard [1,2,3]. The FFF offers numerous advantages such as flap harvesting in a two-team approach to decrease operating time and a single or bi-partitioned septo-cutaneous skin paddle for jaw reconstruction [4]. Jaw reconstruction is in the current focus of research for increasing and reliable results, minimizing complications, and accelerating dental rehabilitation [5,6,7,8]. Virtual surgical planning (VSP) is an essential tool for craniomaxillofacial surgery, from demolition to reconstruction, including orthognathic surgery and oral rehabilitation [9]. Studies showed that VSP facilitates the precise positioning of the bone graft, decreases ischemia time and the entire operating time if the neo-mandible was stabilized with a patient-specific implant (PSI) [10,11,12,13,14]. As an alternative for the stabilization of neo-mandible with PSI, conventional manual-bendable mini-plates and reconstruction plates are still available with each specific advantages and disadvantages [15]. Further improvements can be made by varying the plate design, plate thickness and fixation methods (locking or non-locking screws).

Data about osseous union after jaw reconstruction with FFF concerning the stabilization methods, especially the comparison PSI vs. conventional plates, are rare in the literature. Virtual surgical planning in combination with CAD/CAM plates is a widespread and promising technology. However, the clinical impact of PSI high stiffness fixation methods on bone healing and regeneration is still not well addressed. Therefore, the current study is designed to fill the gap.

This investigation aimed to evaluate the status of osseous union in uni- and poly-segmental mandible reconstructions regarding conventional angle-stable manually bent osteosynthesis plates (2.0 mm) versus PSIs. Furthermore, plate-related complications should be evaluated. The authors hypothesized that the chosen plate system could not influence the osseous union of the inter-segment gaps and related complications. The study focusses on giving detailed answers to the following questions:

Are there differences in incomplete osseous union rates (IOU) according to the used plate type (conventional vs. PSI)?

How is the distribution and frequency of complete and incomplete osseous union regarding FFF’s proximal or distal end?

What is the frequency of plate-related complications (loosening of osteosynthesis, plate exposure)?

## 2. Material and Methods

### 2.1. Study Design and Patient Population

**Figure 1:**
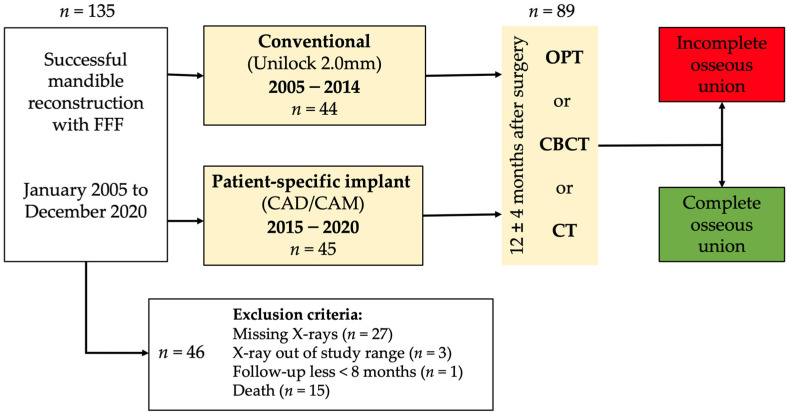
Schematic study design. OPT: Panoramic radiograph; CT: computed tomography; CBCT: cone-beam CT; CAD: computer-aided design; CAM: computer-aided manufacturing; FFF: fibula free flap.

The study was conducted as a monocentric, retrospective study. We enrolled the investigation for immediate and delayed mandible reconstructions. Panoramic radiograph (OPT), computed tomography (CT) scans, and cone-beam CTs (CBCT) of patients who underwent successful FFF for jaw reconstruction from January 2005 to December 2020 were reviewed concerning the osseous union of the intersegmental gaps between mandible and fibula and between fibula segments themselves. The stabilization of the neo-mandible was performed either with conventional plates or individual CAD/CAM-planned patient specific plates after virtual surgical planning. Conventional manually bent plates of 2.0 mm thickness (Unilock 2.0 system, DePuy Synthes CMF, Oberdorf, Switzerland) were compared with laser-melted patient-specific CAD/CAM titanium plates (PSI) with layer thicknesses of 2.0 to 2.5 mm (KLS Martin, Tuttlingen, Germany) in terms of reconstructive features, and complication rates (Figure 1). Conventional plates were placed segmentally, while PSI were applied as continuous plates. Only in the PSI group were cutting guides used for flap harvesting at the donor site and for defining resection planes at the recipient site. In the conventional group, free hand osteotomies were performed for the resection and shaping of the fibula segments. Both conventional and patient-specific plates were almost secured with bi-cortical locking screws in the mandibula. The decision to use locking or non-locking screws for plate anchoring to the FFF was done by a surgeon intraoperatively.

### 2.2. Inclusion and Exclusion Criteria for Study Subjects

All patients who underwent mandible reconstruction (immediately or delayed) with FFF were enrolled in this study. Inclusion criteria were the presence of OPT, CBCT or CT-scan of the jaw 12 ± 4 months after surgery. The minimum follow-up interval was eight months after surgery. A total of *n* = 135 successful FFF were performed over the entire study period, and *n* = 89 patients fulfilled the chosen inclusion criteria. A total number of *n* = 46 cases were excluded from the analysis because of missing X-rays (*n* = 27), death (*n* = 15) and one patient lost to follow-up. Three cases were excluded, because X-ray of the jaws was admitted at least 22 months after surgery and did not match our inclusion criteria. 

### 2.3. Study Parameters and Evaluator Calibration

The patients’ medical records were evaluated for the type of used plate system and complications such as exposed osteosynthesis plates (intraoral and/or extraoral) or material loosening (screw loosening). The reconstruction was categorized according to the classification of Brown et al. [16]. For further evaluation, this was simplified and summarized. Classes I(c) to II(c) correspond to unilateral defects and classes III to IV(c) to anterior, bilateral defects.

**Figure 2:**
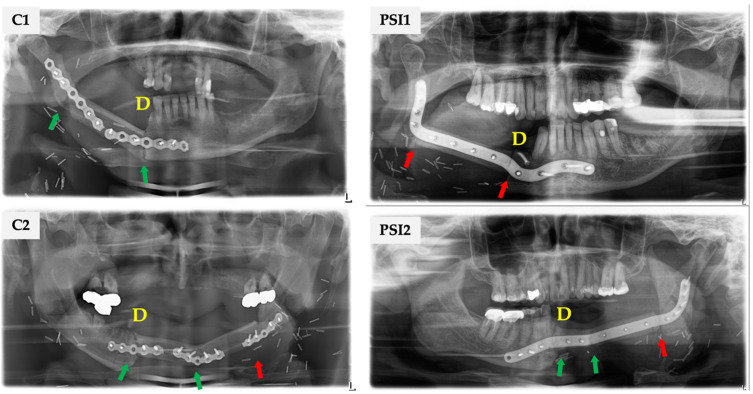
Note: D, distal end of fibula; green arrow: complete osseous union (COU); red arrow: incomplete osseous union (IOU). C1: Uni-segmental, lateral reconstruction of mandible. Two distinct mini-plates Unilock 2.0 system (DePuy Synthes CMF^®^, Raynham, MA, USA) were used for internal fixation. Each junction was addressed with a single plate and locking screws. COU was assessed for the proximal (angle) and distal (anterior corpus) junction. C2: Bi-segmental, bilateral mandible reconstruction, and conventional stabilization. IOU of the proximal transition zone at the posterior corpus region, while the distal (canine) and inter-segment junctions showed COU. PSI1: Uni-segmental, lateral mandible reconstruction and stabilization with PSI (KLS Martin, Tuttlingen, Germany). IOU of the proximal (angle) and distal (anterior corpus) transition zone. PSI2: Bi-segmental, bilateral mandible reconstruction and stabilization with PSI. IOU of the proximal junctions at the posterior corpus region, while the distal (canine) and intersegmental junctions show COU.

The following parameters were collected: age at flap transfer, gender, diagnosis, number of used fibula segments, and ossification status of each gap concerning the orientation of the fibular bone (distal/proximal end). Complete (COU) and incomplete osseous union (IOU) rates were calculated in relation to the related reference group (patients, all gaps at risk, or intersegmental only) of all assessable junctions. The available radiographs (OPT, CT, and CBCT-scans) were analyzed independently for the ossification of each gap by two authors (SS and MK). Ossification in OPT was defined as incomplete (IOU) if the interosseous transition zone appeared less than 50% ossified or as complete (COU) if it appeared more than 50% ossified (Figure 2). In CBCT and CT, axial imaging was considered for assessment. IOU was rated if initial callus formation or a persistent gap between segments or subtotal osseous bridging between corresponding bone cortices or marrow were found. COU was rated if the corresponding cortices were joined without significant gaps. Every gap was assessed by two investigators individually. Any disagreements between the two authors were discussed and judged by a third author (FR) who is a radiologist. If a CT or CBCT was available in addition to an OPT, a higher-quality image source was evaluated for assessment. The evaluated CT scans were initiated within the course of routine follow-up examination. CBCT was mainly performed to plan the insertion of dental implants for oral rehabilitation. Radiological control was performed between the ninth and 14th postoperative months.

### 2.4. Statistical Analyses

Continuous variables were reported using mean, standard deviation, median and interquartile interval (Q1, Q3). Categorical data were recorded as frequencies and percentages. Binary logistic regression statistics was performed. The bivariate analysis included Student’s t-test to compare continuous quantitative variables between both groups (Conventional vs. PSI) after verifying normality (Shapiro–Wilk-test). Chi-square and Fisher tests were performed for categorical variables. Cohen’s Kappa (κ) statistics was calculated to assess the interobserver reliability between SS and MK. *P* < 0.05 was defined as statistically significant. The statistical analysis was performed in collaboration with the Institute of Medical Informatics of Justus Liebig University Giessen using SPSS software version 28 (SPSS Inc., Chicago, IL, USA).

### 2.5. Ethics Statement/Confirmation of Patients’ Permission

The local Ethics Committee of Justus-Liebig University Giessen, Faculty of Medicine, approved the study (AZ35/20 on 25 May 2020). Patients’ permission/consent was not necessary for this retrospective study. The patients consented that their X-ray images could be used anonymously in the publication.

## 3. Results

A total number of *n* = 89 cases (28 females (31.5%), 61 males (68.5%), mean age 58.2 ± 11.3 years, range: 22.8–82.7 years) fulfilled the inclusion criteria for analysis. They were classified in non-VSP, conventional (*n* = 44) and digitally planned PSI (*n* = 45) groups. The parameters age and time of image acquisition (‘Image osseous union’) were verified for normal distribution for both osteosynthesis groups. No statistically significant differences were found. The study sample structure was almost similar in both groups (age, gender, ASA-Score, BMI). The detailed demographic parameters and results are summarized in Table 1.

The main reasons for mandible reconstruction were benign and malign tumors infiltrating the bone (conventional: 95.4% vs. PSI: 91.1%). Medication-related osteonecrosis of the jaw (MRONJ), osteoradionecrosis, and osteomyelitis contributed 4.6% vs. 8.8% to the analyzed groups. The main reconstruction method was immediate (95.5% vs. 86.7%). In the PSI group, 77.8% of defects with classified at least as class II(c) or higher according to the classification of Brown et al. [16], whereas in the conventional group only 63.6% showed a classification of at least II(c). Therefore, a trend for larger and more complex reconstructions can be assumed in the PSI group. Nineteen patients (43.2%) in the conventional group and eight (17.8%) in the PSI group underwent mono-segmental reconstruction. Poly-segmental mandibular reconstruction accounted for 82.2% of the PSI group and 56.8% in the conventional group. Regarding general conditions of the patients there were 61 cases of chronic tobacco abuse (conventional: 72.7% vs. PSI: 64.4%; *p* = 0.495) and 35 of pathologic alcohol usage (conventional: 36.4% vs. PSI: 46.7%; *p* = 0.392).

Complications of the donor and recipient sites are presented in Table 2. In total, there were *n* = 21 cases of plate exposure (conventional: 22.7% vs. PSI: 24.4%; *p* = 1.000) and eleven plate related fixation failures (conventional: 9.1% vs. PSI: 15.6%; *p* = 0.522). Preoperative radiotherapy (RT) occurred only in exceptional cases (conventional: 11.4% vs. PSI: 13.3%) while postoperative RT was frequently used (conventional: 34.1% vs. PSI: 53.3%). The rate of non-irradiated patients has decreased (conventional: 54.5% vs. PSI: 33.3%).

Inter-observer reliability was obtained for the graduation of osseous union. The κ value of 0.934 indicates a good match between the observers. In total, IOU of at least one osteotomy junction between mandibula to fibula or intersegmental in the study sample was found in 35.6% (*n* = 16 out of 45) in the PSI group and in 13.6% (*n* = 6 out of 44) in the conventional group (Table 2). The difference was statistically significant (*p* = 0.017). The difference becomes more apparent when OU was referred to all junctions at risk (conventional: *n* = 6; 5.0% vs. PSI: *n* = 25; 19.4%; *p <* 0.001). In detail, there were *n* = 23 IOU out of assessable *n* = 167 appositions between the native mandibula and the free flap bone, indicating an overall IOU rate of 13.1% (conventional: *n* = 6; 6.8% vs. PSI: *n* = 17; 21.5%; *p* = 0.006). IOU was recorded at the distal junction in *n* = 13 (conventional: *n* = 5 vs. PSI: *n* = 8), at the proximal junction in *n* = 10 patients (conventional: *n* = 1 vs. PSI: *n* = 9). Regarding the intersegmental junctions eight IOU were found in the PSI group while no IOU could be found in the conventional group (conventional: 0/32 vs. PSI: 8/50; *p* = 0.015). Thus, an overall rate of 16.0% IOU was found. Only six patients had IOU of more than one junction in the PSI group.

Ossification status was assessed for the distal and proximal inter-segment junctions and assigned to regions of the mandible (Table 3). The evaluation revealed cumulative rates of IOU at the proximal end of 10.6% (PSI: 19.5% vs. conventional: 2.2%; *p* = 0.009) and the distal end of 16.3% (22.2% vs. 11.4%; *p* = 0.190). In the conventional group only one proximal and five distal cases (11.4%) were recorded. IOU was found to be more frequent at fibula’s distal end. Finally, no preferred region was found in which IOU was observed more frequently. Uni- and poly-segmental mandibular reconstructions were analyzed for the status of osseous union (Table 3). Statistically significant differences for the presence of IOU were found in poly-segmental reconstructions with PSI (*p* = 0.041). The comparison of lateral and anterior reconstructions showed that IOU was found more often after lateral reconstructions with PSI (*p* = 0.016). The relation between the risk factor variables and binary outcomes was assessed by a generalized linear model for binary logistic regression (Table 4). When PSI was used as plate system (OR = 3.494; 95%-CI: 1.216–10.040; *p* = 0.017), plate exposure (OR = 3.173; 95%-CI: 1.105–9.110; *p* = 0.027) and screw loosening (OR = 4.650; 95%-CI: 1.257–17.197; *p* = 0.014) were identified as significant risk factors. Multivariate analysis revealed that only the variables plate system (OR = 3.682; 95%-CI: 1.236–10.966; *p* = 0.019) and plate exposure (OR = 3.389; 95%-CI: 1.118–10.275; *p* = 0.031) are independent risk parameters for incomplete osseous union (Table 5). The model is significant in the Omnibus-Test (*p* = 0.05; Nagelkerkes R^2^ = 0.166). The data were not able to distinguish the reasons for screw loosing (fixation problems vs. osteoradionecrosis). The parameter screw loosing was excluded from multivariate analysis.

## 4. Discussion

Virtual surgical planning (VSP) in combination with custom-made osteosynthesis (patient-specific implants, PSI) has become popular in jaw reconstruction. VSP allows for more extensive, complex and precise reconstructions than the conventional freehand method [17,18,19]. VSP was described for ideal placement of miniplates on a virtual 3D model and adapting these plates in further step on a printed model. The specific advantage of this approach requires a minimum of money and time [20]. However, CAD/CAM implants allow highly precise mono- and poly-segmental shaping and molding of a free fibula flap for ideal jaw reconstruction [21,22,23]. Increased precision of mandibula and maxilla reconstruction, shortened surgery and ischemia time, reduced length of hospital stay, and improved patient outcomes were all common advantages in maxillofacial reconstruction [24,25,26,27,28,29]. However, there are disadvantages concerning the VSP method with PSI such as the prolonged time to surgery [30] and abnormalities in the ossification of the transition zone between the fibula and mandibular stumps. Yang et al. compared *n* = 33 patients (conventional: *n* = 16, PSI: *n* = 17) and found improved accuracy of reconstruction in terms of bilateral mandibular angles and bone grafts, but the accuracy of the osteotomy was similar in both groups. There was no change in intraoperative blood loss, total operation time, or hospital stay [31]. More plate exposure and a higher rate of incomplete osseous union was found for patient-specific plates [32]. Incomplete osseous union (IOU) was described to be more prevalent at the junction zone of the distal fibula segment and in poly-segmental reconstructions [33,34].

### 4.1. Are There Differences in Incomplete Osseous Union (IOU) Rates according to the Used Plate Type (Conventional vs. PSI)?

IOU was found between fibula and mandible stumps and between fibula segments in both osteosynthesis groups (Table 2). The present study revealed an overall IOU rate of 24.7%, which is well comparable to the literature with reported rates between 5% and 45.9% [32,35,36,37,38,39,40]. The study results show in detail a higher IOU rate related to all junctions at risk and referred to the number of patients in the PSI group of 35.6% in comparison to the conventional group with 13.6% (*p* = 0.017). Rendenbach et al. found subtotal osseous union rates in their study sample (*n* = 128) after mandible reconstruction with a rate of 45.9% for PSI in comparison to conventional plating with 33.0% [32].

Significant differences were registered in the present study for IOU rates in poly-segmental (*p =* 0.041), and in lateral (*p =* 0.016) mandibular reconstructions when PSI instead of conventional plate was used. A shielding of mechanical forces due to robust plate systems reduces inter-osteotomy micromotions below a critical functional minimum for bone healing stimulation [41,42,43,44]. In patients with reduced dentition or without postoperative occlusion, and therefore minimal functional loading on the bone, a higher rate of IOU has been reported [32,41]. This underlines the importance of mechanical factors such as strain, pressure, stability, stimulation for bone healing [45]. Thus, one reason for the higher IOU rate in the PSI group is attributed to the increased plate rigidity of CAD/CAM plates [46]. Another difference is the functional force transmission into the bone in the systems used. Conventional plates were placed segmentally, so that each junction (mandible-fibula and fibula-fibula) was addressed with a separate osteosynthesis. The applied masticatory force is thus directed through each link of the “bone chain” and contributes to functional stimulation. In PSI stabilization, the bone segments are adapted to a single plate and introduced forces are absorbed by the continuous load-bearing patient-specific plate. Therefore, the functional stimulus on each bone segment must be lower than in segmentally joined jaw reconstruction. Kreutzer et al. have proposed to replace the PSIs with patient-specific mini-plates [47], and presented recently a feasibility study with *n* = 8 patients [48]. According to their findings and limitations of the small sample size due the feasibility study they concluded, that mandible reconstruction with FFF using patient-specific 3D-printed miniplates is possible and related to good precision, bone healing, and distant soft tissue problems [48]. Alternatively, the planning of predefined separation points in the PSI, which can be divided after the neojaw’s shaping, should be considered. This will increase the force transmission through the “bone chain”, enhances functional stimuli for bone healing, and simplifies later intraoral plate removal if necessary. 

Compared to conventional plating methods, the bone-to-plate contact area is increased in PSIs [46]. Due to the ideal bone surface congruent shape of the PSI, the blood circulation of the periosteum can be impaired by compression [49,50,51]. As a result, bone resorption and screw loosening can occur if non-locking screws are used [52]. In addition to PSI’s technical and biomechanical aspects, additional guides for jaw resection and transplant molding should also be mentioned as a reason for IOU as a result of periosteal trauma and decreased perfusion [53,54]. After VSP for jaw reconstruction, a patient-specific plate had to be applied, either 3-dimensional printed CAD/CAM [17,18] or universal, reusable saw guides [55,56] for resection and donor-site were required. The soft-tissue at the resection site and, in particular, the periosteum of the donor-site disturbs the correct fit of the individual cutting and drill guides. This implicates inaccuracy concerning resection planes and miter cuts. For FFF harvesting it is recommended to leave a preserving muscle cuff of 1 to 2 mm thickness during flap dissection around fibular bone [57], because of the split osseous blood supply of the fibula bone in endosteal and periosteal portions [2,58]. The endosteal blood supply is responsible for over two-thirds of cortical bone blood flow and is the leading player for blood supply in tubular bones [59]. The interruption of the periosteum or endosteum’s integrity appears to impair bone healing in both experimental and clinical studies, but disturbance of either one of these structures does not affect total fracture healing [54,60,61]. Numerous animal studies have shown that the healing potential of cortical bone is dependent on endosteal and periosteal blood flow, which can be impaired due to osteosynthesis [53,54].

### 4.2. How Is the Distribution and Frequency of Complete and Incomplete Osseous Union regarding FFF’s Proximal or Distal End?

IOU was found to be slightly more frequent at fibula’s distal end with a rate of 16.3% (PSI: 22.2% vs. conventional: 11.4%; *p* = 0.190) than proximal 10.6% (19.5% vs. 2.2%; *p* = 0.009). A comparison of the IOU presence concerning the defined transition zones shows no preferred region in which IOU has been found more frequently.

A retrospective study on *n* = 104 mandibular or maxillary resections and subsequent reconstructions with osseous(-cutaneous) free flaps revealed a partial union or non-union rate of 47%. The authors observed significantly higher COU rates between intersegmental junctions (fibula to fibula) than the native mandible [38]. They reported that it was unlikely to detect COU if the intersegmental gap width was broader than 1 mm, and recommended precise shaping to reduce intersegmental gap width to a minimum [38]. The current study observed an IOU rate of 16.0% (*n* = 8) for intersegmental junctions in the PSI group (*n* = 8 out of 50), and no case has been detected in the conventional plating group (*n* = 0 out of 32). This statistically significant (*p* = 0.015) observation must be critically evaluated because of the PSI group’s higher number of poly-segmental reconstructions compared to conventional plates (Table 1).

Bone healing and intersegmental osseous union can be recorded two months after surgery, and structural changes in X-ray become visible, and is dependent on functional stimuli, stress, and shearing [62]. While primary bone healing is dependent on gap width up to 1 mm, broader gaps can heal by secondary bone healing due to callous formation, completing the connection of the two bone segments [63]. Regardless of the accuracy of VSP and individual cutting guides, it is presumable that in the majority of junctions secondary bone healing will occur. Greksa et al. highlighted the impact of periosteal and endosteal microcirculation in bone healing in a case of non-union of the tibia bone after osteosynthesis [64]. When micro-motions occur between the fractured sections of bones covered by the periosteum, endochondral bone repair is the predominant way of bone healing in long bones [51,65]. The importance of the endosteum in bone healing is commonly underestimated due to the periosteum’s essential function [66,67]. The authors hypothesized that the combination of impaired endosteal and periosteal circulation after (multiple) segmentation and decreased mechanical stimulation through the PSI contributes to the higher IOU rates in poly-segmental reconstructions. Visual control for bleeding from the bone marrow is routinely performed after osteotomy of each fibula segment. Clinical experience shows that a significant bleeding does not occur in all cases. What impact this observation has on the osseous union of the junctions remains still unclear.

Following fractures and osteotomies, the size of the interfragmentary gap changes the mechanical environment. Therefore, increasing gap size results in significantly reduced strength and stiffness in mechanical and histological outcomes [62,68,69]. Successful segmental mandible reconstruction with FFF was also reported when mini-plates were used [46,70], but it is recommended not to use them if a radiation therapy is planned [13,71]. *Robey* et al. reviewed *n* = 117 patients after mandible reconstruction with FFF, and compared complication rates when using mini-plates (*n =* 86) and reconstruction plates (*n =* 31). They found a decreased incidence of osteoradionecrosis in the mini-plate group (5% vs. 38%; *p* = 0.02) [43]. This finding must be interpreted cautiously because of the small sample size. However, for PSI there is a leak of data regarding its influence on the incidence of osteoradionecrosis. Shimamoto et al. assessed scatter doses for various dental metals and titanium of the buccal mucosa and different types of radiation therapy in an experimental setting. Except for gold, there were no variations in the scatter doses caused by specific dental metals in the direction of the buccal mucosa in 3D conformal radiation therapy and in intensity-modulated radiation therapy [72].

FFF dissection, multiple osteotomies, and mechanical trauma decrease the endosteal blood flow in the distal segment [73]. Both segmental osteotomies and fixation osteosynthesis plates or screws reduce blood supply to the most distal fibula segment, according to animal investigations on a vascularized pig fibula [73,74]. Segment length and the frequency of proximal osteotomies were associated to distal segment bone perfusion. Longer segments and fewer osteotomies were associated with higher perfusion [33]. These results are in accordance with the observation of a higher complication and non-union rate in the distal segment in poly-segmental mandible reconstructions [34]. Therefore, it is recommended to reduce the number of osteotomies to a minimum, to ensure segmental periosteal circulation and to decrease operating time [75,76]. A human cadaver experiment was conducted to assess the critical segment length. They determined that fibula segments can be revascularized in the recipient bed if they are at least 2 cm long. It can work even in shorter segments, but only partially. Segments smaller than 0.5 cm in length are likely to fail to revascularize and become non-vascularized grafts. Thus, they are recommended not to be used in infected areas or have been subjected to radiation treatment or trauma [77]. During VSP, awareness is necessary to prevent small bone segments to ensure sufficient bone segment perfusion to avoid partial or total flap failure [78]. According to our experience, segments length should not be less than 3.0 cm.

Our results confirmed the findings of other studies [32,33,34], and found only a slightly higher rate of the incomplete osseous union of the distal compared to the proximal gap regarding the entire study population.

### 4.3. What Is the Frequency of Plate-Related Complications (Loosening of Osteosynthesis, Plate Exposure)?

Plate exposure has been recorded in our sample with 23.6% (PSI: 24.4%. vs. conventional: 22.7%), which is almost similar to the literature (PSI vs. conventional: 29.7% vs. 18.7%) [32]. In our study, despite using a composite FFF, conventional and CAD/CAM plates, exposure occurred in all cases intraorally at the canine region around the junction zone of two fibula segments or at the junction fibula to mandible. Dehiscence occurred between the skin paddle and the oral mucosa of the vestibulum or planum buccale. One PSI was exposed through the skin near to the mandible’s angle (Table 2).

Infection, non-union, fistulae, and plate exposure are among the complications linked with mandibulotomies [79]. Some authors argue that bone incision and fixation procedures are likely encouraging factors, while others believe that radiation is involved. Others found no evidence for a single factor, radiation, or fixation type, contributing to a non-union, and recommended careful surgical technique [80]. Plate exposure rates are reported in literature after segmental mandible reconstruction ranging from 4% to 46% [81]. Infection at the surgical site is an independent risk factor for plate exposure [81]. Yao et al. found that postoperative surgical site infection, kind of mandibulectomy defects, and plate profile/thickness were associated with plate exposure in their univariable analysis [81]. In current research concerning removal of PSI following segmental mandible reconstruction, Kreutzer et al. observed that using a FFF septo-cutaneous intraoral skin paddle did not reduce the complication rate resulting in plate removal. They emphasized reviewing critically the indication of a skin paddle to improve donor-side morbidity and subsequent peri-implant soft tissue situation. [47]. Furthermore, the application of resection guides after VSP on the recipient-site requires more extensive hard and soft tissue exposure, and therefore an increasing wound area. It is presumed that a more extended bone exposure is correlated with a higher rate of local infections and plate exposure in the early postoperative course [81].

Plate-related fixation failures were assessed in the present study with 12.3% (PSI: 15.6%. vs. conventional: 9.1%), which is slightly higher than comparable results in the literature (PSI vs. conventional: 8.1% vs. 6.6%) [32]. The PSI group’s higher rate of adjuvant radiation (53.3% vs. 34.1%) has certainly an influence on fixation failure rates but without significance. Based on our clinical experience radiation therapy increases the complications rate postoperatively. This can be confirmed and displayed on irradiated FFF in our study subjects with a screw loosening rate of 42.8% in the PSI group (*n* = 3 out of 7) and 25% in the conventional osteosynthesis group (*n* = 1 out of 4). In two PSI cases, osteoradionecrosis of the FFF occurred, which was reasonable for screw loosening. However, no statistically significant relation could be found regarding screw loosening and radiotherapy, presumably because of the limited number of affected patients. Plate-related problems, however, are nonetheless prevalent when utilizing patient-specific reconstruction plates and may not occur only in patients with significant risk factors such as radiation or poly-segmental reconstructions [48].

Without doubt, technical innovations in smaller plate profiles of reconstructions plates increases biocompatibility and decreases the risk for plate exposure [82,83]. Avoiding bone edges and ridges can help prevent plate exposure. Scarring soft tissue retraction can only be controlled to a limited extent. Therefore, special attention must be paid to the canine region in anterior, poly-segmental reconstructions to design smooth intersegmental transition sections during VSP to decrease the risk of plate exposure. Reducing neo-mandible’s chin prominence in combination with a skin paddle can contribute to a sufficient soft tissue coverage and decreases in our experience the risk of plate exposure. For the beneficial use of a skin paddle different opinions can be found in the literature [47].

Likhterov et al. report their routine fixation procedure with 2.0 or 2.4 locking plates and up to four bi-cortical screws for attaching the plate to the native mandible and one to two mono-cortical screws securing the flap segments to the plate [71]. For PSI fixation, mono-cortical locking [47] or non-locking screws [32] for the fibula flap segment and bi-cortical locking screws to the mandible were described. The advantage of using locking screws for PSI and graft fixation was emphasized in a finite element analysis of a three-segment anterior mandibular reconstruction [49].

In summary, the status of ossification in terms of radiological findings was significantly more frequently assessed as incomplete at the junction between mandible and FFF and between fibula segments in the PSI group compared to the conventional group. These significant differences also remained when comparing the localization (anterior vs. lateral) and the number of segments (uni- vs. poly-segmental) used. Therefore, together with the discussed literature, it can be assumed that the properties of PSI contribute to incomplete ossification.

## 5. Implications

Further studies are necessary to improve PSI’s design and hardware parameters (plate extension, thickness, stiffness, locking vs. non-locking fixation to jaw and graft segments). While bone healing after fracture is well described in the literature, there is a knowledge gap about bone healing of microvascular osseous flaps regarding time interval for the complete osseous union. Additional follow-up trails can be useful to close the data gap regarding the clinical impact of incomplete osseous and its influence on dental rehabilitation with implants.

## 6. Limitations

Limitations of the present study need to be addressed. First, despite the well-known problems of retrospective studies, they allow the covering of a long period of time. Over the study period of 15 years, a core team of four, senior, experienced oral and maxillofacial oncologic and reconstructive surgeons performed immediate jaw reconstructions. Two surgeons overlooked the whole observation time, and the other two observed eight years.

Second, the study focuses on a single image taken around the first year after surgery, and therefore the progression of subtotal ossifications of the free flap segment remains unclear. Although the assessment and classification of ossification were performed independently by two investigators, it was not blinded and was predominantly based on OPTs. Standardized CBCT or CT scans for comparison were not available in all cases. Third, the individual progress of IOU and its clinical impact is left unclear in every single patient’s case. Follow-up evaluation is required to conclude IOU’s impact on necessary surgical treatment and dental rehabilitation.

## 7. Conclusions

Patient-specific implants allow highly accurate poly-segmental, and therefore more complex shaping and molding of a fibula free flap. Incomplete ossification was observed more frequently in the PSI group than in the conventional group. Plate exposure and used plate type were identified as independent risk factors for incomplete osseous union in logistic regression analysis. Previous or adjuvant radiotherapy did not impact incomplete osseous union in the evaluated study sample. This finding should be interpreted carefully as the evidence coming from a limited number of affected patients. Further optimization of the PSI system is required to improve the fibula-jaw bone healing and lowering post-operative complications. To enhance the functional stimulus for ossification it has to be discussed if patient-specific implants can be designed to be thinner, and should be divided into segmental plates. This directs chewing forces through the bone and improves physiological bone remodeling.

## Figures and Tables

**Table 1:** Details of the study sample. Conventional osteosynthesis with Unilock 2.0 system (DePuy Synthes CMF, Oberdorf, Switzerland), CAD/CAM-osteosynthesis with PSI (KLS Martin, Tuttlingen, Germany). IQI: Interquartile interval; MRONJ: Medication-related osteonecrosis of the jaw; PSI: patient-specific implant; Q: quartile; SD: standard deviation.

	Conventional (Unilock 2.0) (*n* = 44)	CAD/CAM (PSI) (*n* = 45)	*p*-Value
Age (years), mean ± SD	58.54 ± 10.46	59.23 ± 12.23	0.777
Image osseous union (months), mean ± SD	11.25 ± 2.52	11.0 ± 2.90	0.665
Follow-up (months), median; IQI (Q1, Q3)	88.0 (35.75, 125.5)	19.0 (12.5, 34.5)	0.001
Gender, *n (*%)			
Male	31 (70.4)	30 (66.7)	
Female	13 (29.6)	15 (33.3)	0.699
Image type			
OPT	29 (65.9)	23 (51.1)	
CBCT	2 (4.5)	2 (4.4)	
CT	13 (29.5)	20 (44.4)	0.321
Pathology, *n* (%)			
Benign tumor	1 (2.3)	5 (11.1)	
Malignant tumor	41 (93.1)	36 (80.0)	
MRONJ	-	1 (2.2)	
Osteoradionecrosis	1 (2.3)	1 (2.2)	
Osteomyelitis	1 (2.3)	2 (4.4)	0.366
ASA, *n* (%)			
1	4 (9.1)	1 (2.2)	
2	22 (50.0)	22 (48.9)	
3	18 (40.9)	21 (46.7)	
4	-	1 (2.2)	0.389
BMI (kg/m^2^), *n* (%)			
<18	3 (6.8)	3 (6.7)	
18 ≥ 25	24 (54.5)	22 (48.9)	
25 ≥ 30	12 (27.3)	14 (31.1)	
30 ≥ 35	3 (6.8)	5 (11.1)	
>35	2 (4.5)	1 (2.2)	0.901
Tobacco abuses, *n* (%)	32 (72.7)	29 (64.4)	0.495
Alcohol abuses, *n* (%)	16 (36.4)	21 (46.7)	0.392
Time of reconstruction, *n* (%)			
Immediate	42 (95.5)	39 (86.7)	
Delayed	2 (4.5)	6 (13.3)	0.266
Brown Classification, *n* (%)			
I(c)	16 (36.4)	10 (22.2)	
II(c)	12 (27.3)	16 (35.6)	
III	16 (36.4)	16 (35.6)	
IV(c)	-	3 (6.7)	0.175
Number of segments			
1	19 (43.2)	8 (17.8)	
2	19 (43.2)	21 (46.7)	
3	6 (13.6)	16 (35.6)	0.010

**Table 2:** Complication rates of the conventional vs. PSI groups. (FFF: free fibula flap; F: fibula; M: mandibula; PSI: patient-specific implant); IOU: incomplete osseous union; COU: complete osseous union; OU: osseous union. Note: In CAD/CAM-PSI group only *n* = 79 junctions in *n* = 45 patients were assessed, because eleven gaps were not interpretable by artifacts of the plate (*n* = 1) and free ending without any contact to the origin mandible (*n* = 10).

	Conventional (Unilock 2.0) (*n* = 44)	CAD/CAM (PSI) (*n* = 45)	*p*-Value
Plate related fixation failures, *n* (%)	4 (9.1)	7 (15.6)	0.522
Plate exposure, *n* (%)	10 (22.7)	11 (24.4)	1.000
Radiotherapy, *n* (%)			
Preoperative	5 (11.4)	6 (13.3)	
Postoperative	15 (34.1)	24 (53.3)	
None	24 (54.5)	15 (33.3)	0.121
OU: M ↔ F and F ↔ F, *n* (%)	44	45	
COU	38 (86.4)	29 (64.6)	
IOU	6 (13.6)	16 (35.6)	0.017
OU: M ↔ F and F ↔ F, *n =* all junctions (%)	120	129	
COU	114 (95.0)	104 (80.6)	
IOU	6 (5.0)	25 (19.4)	<0.001
OU: M ↔ F, *n* = only proximal and distal junctions, (%)	88	79	
COU	82 (93.2)	62 (78.5)	
IOU	6 (6.8)	17 (21.5)	0.006
OU: F ↔ F, *n* = only intersegmental junctions, (%)	32	50	
COU	32 (100.0)	42 (84.0)	
IOU	0	8 (16.0)	0.015
OU uni-segmental reconstruction, *n* (%)	19	8	
COU	16 (84.2)	5 (62.5)	
IOU	3 (15.8)	3 (37.5)	0.215
OU poly-segmental reconstruction, *n* (%)	25	37	
COU	22 (88.0)	24 (64.9)	
IOU	3 (12.0)	13 (35.1)	0.041
OU lateral reconstruction, *n* (%)	28	25	
COU	24 (85.7)	14 (56.0)	
IOU	4 (14.3)	11 (44.0)	0.016
OU anterior reconstruction, *n* (%)	16	20	
COU	14 (87.5)	15 (75.0)	
IOU	2 (12.5)	5 (25.0)	0.346

**Table 3:** Overall incomplete (IOU) versus complete osseous union (COU) was categorized concerning the used plate system and according to the distal or proximal end of the FFF. Co: Condyle; Sco: subcondyle; A: angle; Pc: posterior corpus mandibulae; Ac: anterior corpus mandibulae; C: canine; COU: complete osseous union; IOU: incomplete osseous union; PSI: patient-specific implant.

	Co	Sco	A	Pc	Ac	C	All	IOU-Rate	*p*-Value
Distal junction							(89) 80 *	16.3% *	
COU, conventional	-	5	4	12	10	8	39	-	
IOU, conventional	-	1	1	-	1	2	5	11.4%	
COU, PSI	(9) 0 *	10	3	9	2	4	(37) 28 *	-	
IOU, PSI	-	2	1	1	2	1	8	22.2% *	0.190
Proximal junction							(87) 85 ^‡^	10.6% ^‡^	
COU, conventional	-	5	9	15	6	8	43	-	
IOU, conventional	-	1	-	-	-	-	1	2.2%	
COU, PSI	-	(2) 1 ^‡^	5	9	7	11	(34) 33 ^‡^	-	
IOU, PSI	-	1	2	(2) 1 ^‡^	2	2	(9) 8 ^‡^	19.5%	0.009

Note: * When resection of the mandible including the condyle was done, the fibula’s distal end was shaped into a neo-condyle. Therefore, no osseous union can be expected for this region and *n* = 9 were excluded from the statistical analysis. ^‡^ Proximal region in PSI-group was in two cases not interpretable, because of artifacts by the plate (*n* = 1) and free ending without any contact to the origin mandible (*n* = 1).

**Table 4:** Univariate analysis of patient, surgery, and complication-related parameters on the incomplete osseous union. 95%-CI: 95%-confidence interval; OR: Odds ratio; PSI: patient-specific implant; SD: standard deviation; WHD: wound healing disorder.

		Incomplete Osseous Union			
		Yes, *n* (%)	No, *n* (%)	*p*-Value	OR [95%-CI]
Patient-related parameter					
Age, years (Mean ± SD)		58.91 ± 12.70	58.79 ± 10.92	0.967	1.001 [0.959; 1.045]
Gender	Male	14 (63.6)	47 (70.1)	0.568	0.745 [0.270; 2.053]
	Female	8 (36.4)	20 (29.9)		
Tobacco	Yes	16 (72.7	45 (67.2)	0.626	1.304 [0.448; 3.793]
	No	6 (27.3)	22 (32.8)		
Alcohol	Yes	9 (40.9)	28 (41.8)	0.942	0.964 [0.362; 2.566]
	No	13 (59.1)	39 (58.2)		
ASA-Score ≥ 3	Yes	8 (36.4)	31 (46.3)	0.417	1.958 [0.428; 8.959]
	No	14 (63.6)	36 (53.7)		
BMI (Mean ± SD)		24.81 ± 3.71	24.54 ± 4.90	0.811	1.013 [0.913; 1.124]
Surgery related parameter					
Operation duration, minutes (Mean ± SD)		533 ± 132	512 ± 91	0.395	1.002 [0.997; 1.007]
Reconstruction	Immediate	19 (86.4)	62 (92.5)	0.380	1.958 [0.428; 8.959]
	Delayed	3 (13.6)	5 (7.5)		
Plate system	Conventional	6 (27.3)	38 (56.7)	0.017	3.494 [1.216; 10.040]
	PSI	16 (72.7)	29 (43.3)		
Fibular segments	1	6 (27.3)	21 (31.3)	0.800	0.919 [0.479; 1.765]
	2	15 (54.5)	28 (41.8)		
	3	4 (18.2)	18 (26.9)		
Reconstruction site	Unilateral	15 (68.2)	39 (58.2)	0.406	0.650 [0234; 1.803]
	Bilateral	7 (31.8)	28 (41.8)		
Radiotherapy	None	8 (36.4)	31 (46.3)	0.432	1.231 [0.733; 2.068]
	Preoperative	3 (13.6)	8 (11.9)		
	Postoperative	11 (50.0)	28 (41.8)		
Complication					
Plate exposure	Yes	9 (40.9)	12 (17.9)	0.027	3.173 [1.105; 9.110]
	No	13 (59.1)	55 (82.1)		
Plate related complication (screw loosening)	Yes	6 (27.3)	5 (7.5)	0.014	4.650 [1.257; 17.197]
	No	16 (72.7)	62 (92.5)		

**Table 5:** Multivariate analysis of significant risk factors on the incomplete osseous union in univariate binary logistic regression.

Parameter	*p*-Value	OR	95% CI	
Plate system	0.019	3.682	1.236	10.966
Plate exposure	0.031	3.389	1.118	10.275

## Data Availability

The data presented in this study are available on request from the corresponding author.

## Abbreviations

A: angle; Ac, anterior corpus mandibulae; BMI, body mass index; C, canine; Co, condyle; COU, complete osseous union; F, fibula; FFF, fibula free flap; IOU, incomplete osseous union; IQI, Interquartile interval; M, mandibula; MRONJ, medication-related osteonecrosis of the jaw; OU, osseous union; Pc, posterior corpus mandibulae; PSI, patient-specific implant; Q, quartile; Sco, subcondyle; SD, standard deviation; VSP, virtual surgical planning.
